# Roles of polyunsaturated fatty acids, from mediators to membranes

**DOI:** 10.1194/jlr.R120000800

**Published:** 2020-06-02

**Authors:** Takeshi Harayama, Takao Shimizu

**Affiliations:** * Department of Biochemistry and National Centre of Competence in Research in Chemical Biology, University of Geneva, CH-1211 Geneva, Switzerland; † Department of Lipid Signaling, National Center for Global Health and Medicine, Shinjuku-ku, Tokyo 162-8655, Japan and Department of Lipidomics, Graduate School of Medicine, University of Tokyo, Bunkyo-ku, Tokyo 113-0033, Japan

**Keywords:** membrane biology, eicosanoids, G protein-coupled receptors, membrane biophysics, glycerophospholipids

## Abstract

PUFAs, such as AA and DHA, are recognized as important biomolecules, but understanding their precise roles and modes of action remains challenging. PUFAs are precursors for a plethora of signaling lipids, for which knowledge about synthetic pathways and receptors has accumulated. However, due to their extreme diversity and the ambiguity concerning the identity of their cognate receptors, the roles of PUFA-derived signaling lipids require more investigation. In addition, PUFA functions cannot be explained just as lipid mediator precursors because they are also critical for the regulation of membrane biophysical properties. The presence of PUFAs in membrane lipids also affects the functions of transmembrane proteins and peripheral membrane proteins. Although the roles of PUFAs as membrane lipid building blocks were difficult to analyze, the discovery of lysophospholipid acyltransferases (LPLATs), which are critical for their incorporation, advanced our understanding. Recent studies unveiled how LPLATs affect PUFA levels in membrane lipids, and their genetic manipulation became an excellent strategy to study the roles of PUFA-containing lipids. In this review, we will provide an overview of metabolic pathways regulating PUFAs as lipid mediator precursors and membrane components and update recent progress about their functions. Some issues to be solved for future research will also be discussed.

The importance of PUFAs in health and disease gained general attention, as exemplified by the belief that eating more ω-3 PUFAs is good for health. PUFAs are fatty acids that have two or more double bonds, which can be illustrated as XX:Yω-Z (XX, Y, and Z are carbon number, double bond number, and the position of the first double bond from the methyl end, respectively). They cannot be synthesized endogenously in mammals, except for mead acid (20:3ω-9), which is produced under PUFA deficiency (1). Therefore, PUFAs are essential nutrients that have to be obtained from the diet. While important PUFAs, such as AA (20:4ω-6) and DHA (22:6ω-3), can be directly taken up from the diet, they can also be converted from other PUFAs endogenously. The liver has a major contribution in this process, where dietary linoleic acid (LA; 18:2ω-6) and α-linolenic acid (18:3ω-3) are metabolized into other PUFAs by desaturases, elongases, and peroxisomal β-oxidation (2) (**Fig. 1A**). Peroxisomes are critical for the final step of DHA synthesis, and their dysfunction leads to the accumulation of the otherwise minor intermediate PUFA, tetracosahexaenoic acid (24:6ω-3) (3). Analysis of mice lacking elongases or desaturases revealed PUFA functions in brain, metabolic tissues, reproductive organs, and blood cells (4–13). While some of the phenotypes of these mice were seen only when fed PUFA-deficient diets, others, such as the lean phenotype of *Fads1*-deficient mice (14), were observed even when the diets were PUFA sufficient (7). This supports the importance of obtaining sufficient levels of PUFAs both from the diet and from endogenous conversion. Importantly, the possibility to rescue the phenotypes by dietary intervention was dependent on which PUFA to give, clearly demonstrating the nonredundancy in their functions (**Table 1**). The importance of PUFAs has also been analyzed by feeding them in cell culture, revealing important roles in osteogenesis (15) and mechanosensing (16), among many others. While such in vivo and in cellulo experiments support the biological importance of PUFAs, understanding the molecular mechanisms of PUFA functions remains challenging.

**Fig. 1.**
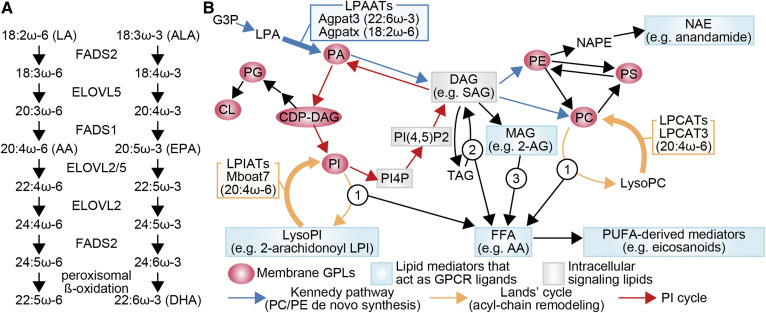
Synthesis and metabolism of PUFAs. A: PUFA conversion from the essential fatty acids, LA and α-linolenic acid (ALA), by the indicated enzymes. FADS2 is proposed to desaturate other positions as well (115), which is not depicted here. “XX:Yω-Z” illustrates a PUFA with XX carbons, Y double bonds, and the first double bond at the Zth carbon from the methyl end. Note the absence of inter-conversion between ω-6 and ω-3 PUFAs. B: Synthesis of GPLs from glycerol 3-phosphate (G3P), and generation of lipid mediators thereof. Arrows with numbers illustrate steps where free fatty acids (FFAs) are generated (arrow 1, PLA_2_; arrow 2, TAG lipase; and arrow 3, MAG lipase), which can be further used to generate PUFA-derivatives. For simplicity, acyl-chain remodeling (Lands’ cycle) is illustrated only for PC and PI, but the pathway is functional for other GPLs as well. The LPLATs important for PUFA incorporation are illustrated. Although the presence of a LPAAT important for LA accumulation is suggested (72), the responsible gene is unknown (indicated as “x” here). Cofactors and byproducts of enzymatic reactions are omitted. AGPAT3 and MBOAT7 are also termed LPAAT3 and LPIAT1, respectively. CDP-DAG, cytidine diphosphate-diacylglycerol; CL, cardiolipin; DAG, diacylglycerol; LPA, lysoPA; MAG, monoacylglycerol; NAE, N-acylethanolamine; NAPE, N-acyl PE; PI(4,5)P2, PI 4,5-bisphosphate; PI4P, PI 4-phosphate; SAG, stearoyl-arachidonoyl glycerol; TAG, triacylglycerol.

**TABLE 1.** Phenotypes of mice lacking the enzymes of Fig. 1A (not exhaustive) or LPLATs involved in the incorporation of PUFAs into membrane GPLs

Gene	Tissue	PUFAs Decreased	PUFAs Increased	Phenotype	Rescued By	Not Rescued By
Elovl2	Testis	22:5ω-6, 22:6ω-3, 24:5ω-6, 26:5ω-6, 28:5ω-6, 30:5ω-6	20:4ω-6, 22:4ω-6	Sterility	—	22:6ω-3
	Liver	22:5ω-6, 22:6ω-3	20:4ω-6, 20:5ω-3, 22:4ω-6, 22:5ω-3	Prevention of hepatic steatosis	22:6ω-3	—
	Liver	—	—	High nuclear SREBP-1c	—	22:6ω-3
	Macrophages	—	—	Changes in inflammatory profiles	22:6ω-3	—
Elovl5	Liver	20:4ω-6, 20:5ω-3, 22:6ω-3	18:2ω-6, 18:3ω-3, 18:4ω-3	Hepatic steatosis	20:4ω-6, 22:6ω-3	—
Fads1	Whole body	20:4ω-6	20:3ω-6	Lethality	20:4ω-6	—
Fads2	Platelets	—	—	Reduced thromboembolism	20:4ω-6	—
	Ovary	20:4, 22:6, 24:5	18:2, 20:2	Sterility	20:5ω-3 + 22:6ω-3	—
	Testis	20:4, 22:5, 22:6, 24:5, 26:5, 28:5, 30:5	18:2	Sterility	20:5ω-3 + 22:6ω-3	—
	Skin, intestine	—	—	Ulcer formation	20:4ω-6	—
	Testis	20:4ω-6, 20:5ω-3, 22:4ω-6, 22:5ω-6, 22:6ω-3, 26:5ω-6, 28:5ω-6, 30:5ω-6	20:3(Δ7,11,14)	Sterility	22:6ω-3	20:4ω-6 (partial)
	Liver	20:4, 22:6	18:2, 20:3	Hepatic steatosis	20:4ω-6	22:6ω-3
	Whole body	—	—	Weight loss	20:4ω-6	22:6ω-3
	Brain	20:4ω-6, 22:4ω-6, 22:6ω-3	18:2ω-6	Decreased brain functions	22:6ω-3	20:4ω-6 (partial)
Agpat3/Lpaat3	Testis	22:6ω-3	—	Sterility	—	—
	Retina	22:6ω-3	—	Blindness	—	—
Lpcat3	Small intestine	18:2ω-6, 20:4ω-6	22:4ω-6, 22:5ω-6, 22:6ω-3	Neutral lipid overaccumulation	—	—
	Small intestine	18:2, 18:3, 20:4	—	Neutral lipid overaccumulation	—	—
	Intestinal stem cells	18:2, 20:4	—	Cholesterol overproduction, overproliferation	—	—
	Embryonic liver	18:2ω-6, 20:3ω-6, 20:4ω-6	22:4ω-6, 22:5ω-6, 22:6ω-3	Neutral lipid overaccumulation	—	—
	Liver	20:4	—	Reduced neutral lipid secretion	—	—
	Liver	20:4	—	Reduced nuclear SREBP-1c	—	—
	Hematopoietic cells	20:4, 20:5	22:4	Increased cholesterol, promotion of atherosclerosis	—	—
	Myeloid cells	20:4	—	Altered cytokine profile	—	—
Mboat7/Lpiat1	Brain	20:4	—	Brain malformation, fatty liver	—	—

Only phenotypes that were tested for rescue by dietary PUFAs are listed in the case for elongases and desaturases.

PUFAs have two major modes of action: precursors of signaling lipids (lipid mediators) and building blocks of membrane lipids (17). It is thus important to recognize the pleiotropic functions of PUFAs and dissect their modes of action. This necessity made PUFA research an exciting multidisciplinary field at the interface of biology, biophysics, and chemical biology. Here, we will highlight the complex metabolism of PUFAs, the investigation of which elucidated PUFA functions, as lipid mediators and in membranes.

## PUFA-DERIVATIVES IN SIGNALING

**Fig. 2.**
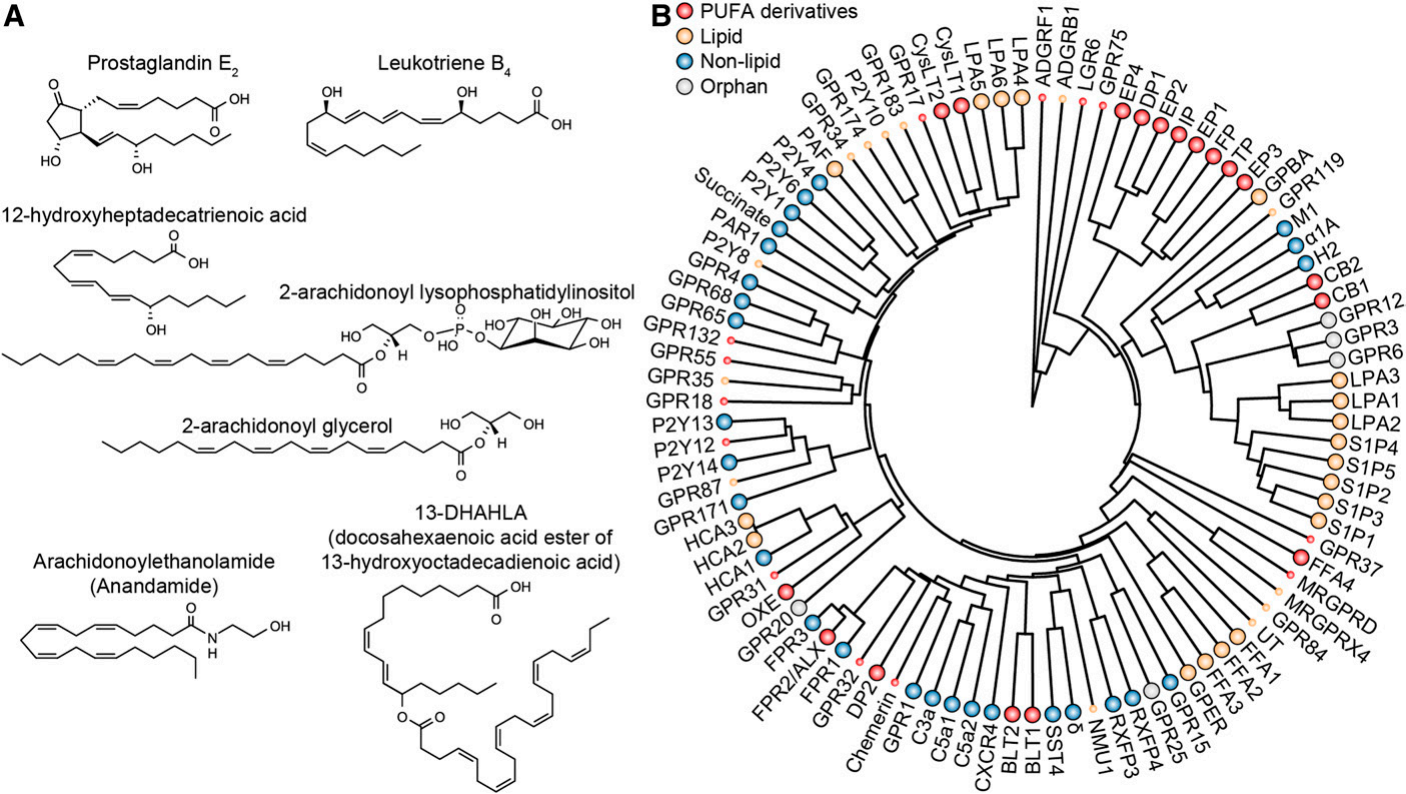
PUFA-derivatives as ligands and their receptors. A: Structures of selected PUFA-derivatives. Prostaglandin E_2_ (ligand for EP1-EP4), leukotriene B_4_ (ligand for BLT1), and 12-hydroxyheptadecatrienoic acid (ligand for BLT2) are metabolites of AA. 2-Arachidonoyl lysophosphatidylinositol (ligand for GPR55), 2-arachidonoyl glycerol, and anandamide (both ligands for CB1 and CB2) contain AA in the esterified form. 13-DHAHLA was recently discovered and its receptor is unknown. B: Phylogenetic tree of selected GPCRs, with emphasis on those that use lipids as ligands. The nomenclature and phylogenetic tree analysis of receptors is based on GPCRdb (https://gpcrdb.org). Smaller symbols represent receptors of which the name does not reflect the proposed lipid ligand. Replication studies are especially important for these receptors. Note that some of the proteins not classified as “PUFA-derivative” receptors can still use PUFA-derivatives as ligands (e.g., some lysophosphatidic acid receptors can sense PUFA-containing molecules), although they do not sense PUFA-derivatives exclusively.

Multiple lipid mediators are produced downstream of glycerophospholipid (GPL) synthesis (Fig. 1B). They include metabolites of PUFAs (e.g., eicosanoids, docosanoids) and lipids with esterified PUFAs [e.g., 2-arachidonoylglycerol (2-AG)], collectively termed PUFA-derivatives here (**Fig. 2A**).

### Diversity of PUFA-derivatives

AA is mainly esterified at the *sn-*2 position of GPLs, such as PC, PE, PS, phosphatidylglycerol, and PI. PI is especially rich in AA (with stearic acid at the *sn-*1 position), while other GPLs have more diverse acyl-chains (18). After being released from GPLs by phospholipase A_2_ (PLA_2_) enzymes (Fig. 1B, arrow 1), AA is converted into more than a hundred different lipid mediators (eicosanoids) by cyclooxygenases, lipoxygenases, cytochrome P450 enzymes, and specific synthases (17). Among PLA_2_s, cytosolic PLA_2_α (cPLA_2_α) causes translocation from cytosol to perinuclear membranes upon various stimuli and is especially important for eicosanoid production (19). Analyses of cPLA_2_α using hydrogen/deuterium exchange mass spectrometry and molecular dynamics revealed how the enzyme uses its C2 domain to bind membranes upon calcium binding (20, 21), to which atomistic details were brought by a recent structural analysis of the C2 domain complexed with a PC membrane (19). In addition to perinuclear membranes, cPLA_2_α localizes to the base of primary cilia, where it releases AA to regulate ciliary trafficking of Smoothened, the signal transducer of the Sonic Hedgehog pathway (22). Mice lacking cPLA_2_α have drastic decreases in eicosanoids and are protected from disease models that include anaphylaxis, asthma, and experimental allergic encephalomyelitis (17). However, they also display abnormalities in labor and synaptic plasticity, demonstrating the pleiotropy of eicosanoid functions (23, 24). The implication of other phospholipases in the release of PUFAs from GPLs is well recapitulated in another review (25). While AA release from GPLs has been studied intensively, other sources of AA for eicosanoid synthesis have been documented, such as triglycerides and 2-AG (26, 27) (Fig. 1B, arrows 2 and 3). Eicosanoids produced downstream of the hydrolysis of 2-AG (which is also a lipid mediator) by monoacylglycerol lipase are especially important in the brain, with implications in neuroinflammation and fever responses (27, 28). Blockade of this pathway reduces eicosanoid production and ameliorates a mouse model of Alzheimer’s disease (29). This pathway is also active outside the brain, as has been shown in a model of hepatic injury (30). PI, being rich in AA, is a good source of 2-AG, and might feed efficiently into this pathway (Fig. 1B).

Eicosanoids are only one part of the diverse repertoire of PUFA-derivatives; other PUFAs are converted into lipid mediators through similar pathways. Those generated from the ω-3 PUFAs, EPA and DHA, are generally regarded as pro-resolving lipid mediators, in contrast to the pro-inflammatory eicosanoids (31, 32). This view is oversimplified though, and some ω-3 PUFA-derivatives promote allergic responses while the eicosanoid lipoxin A_4_ is pro-resolving (31–33). Recent investigations revealed important roles of the secreted PLA_2_ group 2D (PLA2G2D) in the release of PUFAs, including ω-3 PUFAs, from extracellular GPL substrates to generate pro-resolving lipid mediators. PLA2G2D deficiency reduces the production of pro-resolving lipid mediators in the lymph nodes and delays the resolution of contact dermatitis in mice (34). In addition, DHA released by M2-type macrophage-derived PLA2G2D in adipose tissues promotes the generation of beige adipocytes (35). DHA acts as a ligand for free fatty acid receptor 4, which is expressed in primary cilia of preadipocytes and regulates adipogenesis (36). Thus, the ω-3 PUFA DHA has various effects on adipocyte functions. Together with its anti-inflammatory function on macrophages (37), the pleiotropic roles of DHA as a lipid mediator contribute to metabolic health.

Lipid mediators containing esterified PUFAs are also numerous, such as the cannabinoid receptor ligands 2-AG and anandamide (*N*-arachidonoylethanolamine). They mediate retrograde signaling in neuronal synapses and regulate appetite and pain, while having peripheral functions in the immune system as well (38, 39). Another example is 2-arachidonoyl LPI, which is synthesized from PI via the action of phospholipase A_1_ (40). One of its functions is to regulate lymphocyte migration as a ligand for the G protein-coupled receptor (GPCR) GPR55 (41). In addition to this already exhaustive list, novel PUFA-derivatives and synthetic pathways are continuously discovered, such as the hemiketal eicosanoids and fatty acid esters of hydroxy fatty acids (FAHFAs) (42, 43).

### Signaling by PUFA-derivatives

PUFA-derivatives signal through their receptors, which include GPCRs, ion channels, and nuclear receptors (the last two tend to be more promiscuous). So far, many GPCRs are proposed as PUFA-derivative receptors (Fig. 2B), but conflicting results are often reported between studies using different assays (44, 45), and some might be invalidated in the future. In addition, the presence of multiple receptors for the same ligand [e.g., four prostaglandin E_2_ receptors (46)] or the presence of nonlipid ligands for proposed lipid receptors [e.g., GPR132, also termed G2A, reported to be a proton sensor (47) and a receptor for 9-hydroxyoctadecadienoic acid (48); chemerin being a protein ligand for the proposed receptor of resolvin E_1_ (49)] should be taken into account when considering the functions of PUFA-derivatives. For example, GPR37 is proposed as the receptor for DHA-derived neuroprotective and anti-inflammatory protectin D1 (50), while the peptide prosaptide also acts through the same receptor and has similar neuroprotective roles (51). Therefore, it will be important for future studies, especially for PUFA-derivatives that do not have a long history of research, to investigate whether the effects of receptor blockade correspond to those of interference with ligand synthesis.

For PUFA-derivatives having well-validated receptors, such as the eicosanoid prostaglandin E_2,_ genetic disruption or inhibitors can be used to investigate in vivo functions. Prostaglandin E_2_ has been implicated in colon cancer through multiple genetic studies, with consistent phenotypes between mutant mice of synthetic enzymes (52, 53) and receptors (54). In a recent study, single-cell RNA sequencing was used to identify a rare fibroblast population producing prostaglandin E_2_, which promotes tumor initiation by activating Yap nuclear localization in stem cells that are in proximity (55). In the same study, fibroblast-produced prostaglandin E_2_ was shown to be part of a regenerative program after intestinal damage, thereby revealing the correct biological functions of this lipid mediator. With reliable information about synthetic enzymes and receptors, single-cell RNA sequencing approaches might be used similarly in different contexts to identify PUFA-derivative functions.

Our understanding of PUFA-derivative signaling advanced especially in the context of structural biology. The number of reported GPCR structures is expanding, such as the crystal structures of leukotriene B_4_ receptor BLT1 and the prostaglandin D_2_ receptor DP2 (56, 57). The structure of prostaglandin E_2_ receptor EP3 was solved in the presence of its endogenous ligand, revealing critical insights into ligand recognition (58). Molecular docking and molecular dynamics simulations have also been used to investigate the recognition of endogenous ligands for other receptors (59, 60). The structure of the cannabinoid receptor CB1 has been solved in multiple states, bound to inverse agonists, antagonists, agonists, and allosteric modulators or in complex with trimeric G_i_ protein, revealing the structural rearrangements upon ligand binding that lead to signaling events (59, 61–64). The structural characteristics of some GPCRs (e.g., DP2, EP4, TP, and LPA6), such as the presence of an occluded extracellular surface and side openings between transmembrane helices, suggested that the amphiphilic lipid ligands reach the receptor by lateral diffusion in the plasma membrane rather than directly from outside of the cells (57, 60, 65, 66). On the other hand, BLT1 ligand binding pocket is open on the extracellular surface (56), thus different lipid ligands reach their receptors in distinct ways. It will be interesting to understand how these different receptor-accessing modes affect the functions of PUFA-derivatives, such as their effective concentrations, the recognition of their concentration gradients, and their autocrine or paracrine behavior.

To conclude, signaling by PUFA-derivatives is complex due to their diversity, the presence of multiple synthetic sources, and the multiple receptors of a single ligand. Nevertheless, genetic studies of enzymes and receptors unveiled various roles of PUFA-derivatives (17, 46, 67, 68), and structural analyses revealed atomistic insights of their functions. Methodological advances are ongoing to detect lipid mediators comprehensively (69) and to unveil the spatiotemporal regulation of receptor activation in vivo (70). It will be important to solve controversies regarding the assignment PUFA-derivative receptors, and researchers are encouraged to publish more replication studies (both positive and negative). With more reliability in receptors, we will be able to understand the degree to which PUFAs affect pathophysiological conditions as lipid mediator precursors, which might reveal novel therapeutic targets.

## MEMBRANE PUFAS

### Regulation of PUFA-GPLs

While dietary uptake affects whole-organism PUFA supply, tissue-specific functions of GPLs containing PUFAs (PUFA-GPLs) require factors that regulate their tissue distribution. The incorporation of PUFAs at the GPL *sn-*2 position, their main localization, is catalyzed by lysophospholipid acyltransferases (LPLATs) (Fig. 1B, bold arrows). These enzymes use lyso-GPLs and acyl-CoAs as substrates. During GPL de novo synthesis, lysophosphatidic acid acyltransferases (LPAATs) are the LPLATs that acylate the *sn-*2 position and generate the common precursor PA, which is further converted to other GPLs. Acyl-chains of individual GPLs can be remodeled in the “Lands’ cycle,” where one acyl-chain is removed and another one is reincorporated by other LPLATs (71, 72).

At least for PC (the most abundant GPL), expression levels of LPLATs contribute to tissue differences in PUFA-GPL levels, where LPAATs and other LPLATs have different roles. LPAATs enable the accumulation of LA and DHA in PC (and possibly to other GPLs), while AA is mainly incorporated during the Lands’ cycle (72, 73). This conclusion is based on the correlation between PC acyl-chain composition and LPLAT substrate selectivity in various tissues (72), which was later validated in part by genetic studies (72–75). Indeed, disruption of the DHA-preferring AGPAT3 (also termed LPAAT3) drastically reduces DHA-containing GPLs (DHA-GPLs) (74, 75), while loss of the Lands’ cycle LPLATs, LPCAT3, and MBOAT7 (also termed LPIAT1) lowers AA levels in PC, PE, PS, and PI, respectively (73, 76). Enzyme activity measurements suggest the presence of LPAAT enzyme(s) regulating LA levels (72), but the molecular identity of the enzyme(s) remains to be identified. Thus, LPLATs regulate PUFA levels and tissue distribution, with clearly distinct contribution depending on the individual PUFA. High AA levels in PI might assist the PI cycle (Fig. 1B), where the PI head group is sequentially modified for signaling purposes, because some enzymes of the cycle prefer AA-containing substrates (18).

While it is clear that LPLATs have a strong impact on the levels of PUFAs found in GPLs of different tissues, factors that affect tissue supply of PUFAs also exist. For example, MFSD2A is a lipid transporter required for DHA accretion in the brain. Importantly, MFSD2A does not transport free DHA, but rather lyso-PC that contains DHA (DHA-lyso-PC) (77). Thus, DHA-lyso-PC in the bloodstream is a critical source for brain DHA. While such a selectivity was already reported (78), the identification of MFSD2A provided clear molecular mechanisms. Strategies for improved DHA delivery to the brain are developing based on this discovery (79, 80), which might be tested for a therapeutic potential in neuronal diseases where DHA levels are decreased. Another factor affecting brain DHA levels is PE methyltransferase, which converts PE into PC in the liver (81). PE methyltransferase has preference to DHA-containing PE (DHA-PE). Thus, DHA delivery to the brain is a multistep process, requiring the conversion of DHA-PE into DHA-PC in the liver, and then its conversion into DHA-lyso-PC, which is finally taken up by MFSD2A. The mechanisms regulating DHA-PE levels and the enzymes required for DHA-lyso-PC synthesis remain to be clarified. For the latter, phospholipase A_1_ activity would be required, for which hepatic lipase and endothelial lipase are good candidates (80). The identification of other factors affecting tissue-specific PUFA uptake will be of great relevance. For example, fatty acid transporter protein 2 was recently found to affect AA uptake in neutrophils (82). It will be important to investigate how specifically this transporter and other related ones affect PUFA levels in various tissues.

### Mechanisms of PUFA functions in membranes

**Fig. 3.**
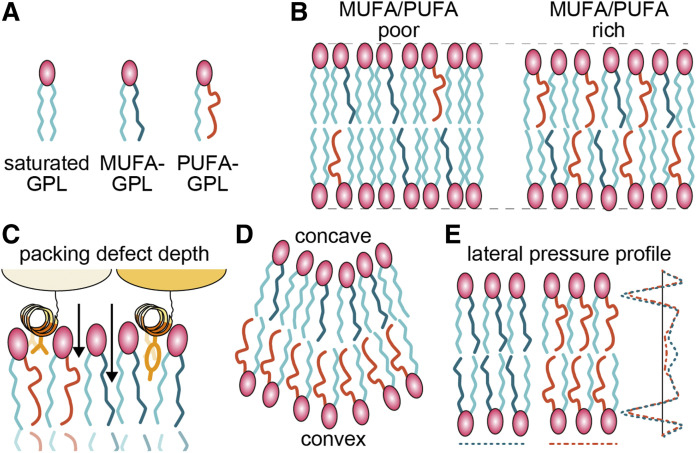
Physical properties of membranes affected by PUFA-containing GPLs. A: Color coding of GPLs. In this illustration, all the GPLs have a saturated acyl-chain at the *sn-*1 position, as is mainly seen in cells. B: Membranes with fewer unsaturated GPLs tend to be thicker (and less fluid) than those with more unsaturated GPLs, due to the higher order in the acyl-chains. Here, we assume the presence of unsaturated GPLs in both cases, to a level that prevents the formation of a gel phase. C: Unsaturated GPLs promote the formation of packing defects, which are water-accessible voids in membrane hydrophobic regions. Packing defects are more abundant in bent membranes not depicted here. The depth of packing defects tends to be shallower in the presence of PUFA-GPLs (arrows). Packing defects promote membrane binding of protein amphipathic helices, and their depth affects the selectivity of this interaction. Shallower defects accommodate amphipathic helices with less bulky hydrophobic side chains. D: PUFA-GPLs, when present in the convex leaflet of a bent membrane, decrease bending rigidity. E: Acyl-chains of GPLs affect the depth-dependent pressure profile exerted on neighboring molecules (lateral pressure profile), which can affect transmembrane protein functions.

PUFAs in GPLs have a higher structural plasticity than saturated fatty acids or MUFAs and can adopt highly kinked shapes. Consequently, PUFA-GPLs increase membrane disorder and affect their physical properties (83) (**Fig. 3A**–D). Because MUFAs also induce disorder, we need to discuss PUFA functions with care and evaluate whether their effects on membrane physical properties differ from those of MUFAs in a biologically relevant manner. Also, PUFA-GPLs affect many physical properties simultaneously, and it remains difficult to know which ones, or which combinations of them, are important.

#### Fluidity.

PUFA-GPLs are thought to increase membrane fluidity. While this is often proposed as a mechanism for PUFA functions, it is rarely shown quantitatively that the gain of fluidity (here defined as the speed of molecules’ diffusion and rotation) by PUFAs (compared with MUFAs) is sufficient to explain a phenomenon. The role of PUFAs in fluidity regulation has even been questioned (84). A recent study combined theoretical and experimental approaches to demonstrate that bacterial respiration is a diffusion-limited process and that MUFAs have quantitatively enough impact on this process (85). In another study, researchers combined reconstitution assays, biophysical measurements, and molecular dynamics simulations, and found that the yeast sensor for membrane unsaturation and transcription factor Mga2 is not affected by fluidity per se but rather by depth-dependent membrane packing (86). This study is a good example of why it is oversimplified to explain all the functions of unsaturated lipids through membrane fluidity. Therefore, future investigations about PUFA functions that rely on fluidity should be conducted carefully, as was done in the aforementioned studies.

#### Thickness.

The disorder induced by PUFA-GPLs and MUFA-GPLs makes membranes thinner when compared with saturated fatty acid-GPLs (Fig. 3B). Membrane thickness affects the localization and function of transmembrane proteins, which is a possible mechanism for PUFA functions (87). It should be noted that highly unsaturated PUFAs are often longer than MUFAs, thus PUFA-GPLs do not necessarily make membranes thinner than MUFA-GPLs do (88).

#### Packing defects.

Packing defects are water-accessible voids in the hydrophobic part of membranes, which accumulate in the presence of unsaturated lipids or conical lipids, or upon membrane bending (Fig. 3C). Protein amphipathic helices can interact with membranes through packing defects, which is used as a strategy to sense vesicles with different lipid compositions (89). While GPLs with MUFAs or PUFAs both generate packing defects, PUFA-GPLs make shallower ones, due to their structural flexibility (90). The depth of packing defects affects the binding of amphipathic helices with various side chains; thus PUFA-GPLs might regulate protein-membrane interactions.

#### Bending rigidity.

PUFA-GPLs make membranes more flexible than MUFA-GPLs (91) (Fig. 3D). This is attributable to the ability to reduce packing defects upon bending (and possibly to thickness). Thus, PUFAs affect processes where membrane bending occurs, as has been shown for endocytosis (90). Bending rigidity is a plausible mechanism for in vivo PUFA functions, as will be explained later. Importantly, only PUFA-GPLs present at the convex leaflet of a bent membrane reduce rigidity (91), which is consistent with the asymmetrical distribution of PUFA-GPLs in the plasma membrane (92), if considering endocytosis.

#### Protein conformation.

Lateral pressure profiles illustrate local forces that are applied, for example to a membrane protein, depending on the depth in the membrane. Double bond positions in GPLs affect this property, which can change membrane protein conformation (93) (Fig. 3E). The conformational flexibility of PUFAs could also assist dynamic changes in membrane protein conformation (83).

#### Chemical properties.

Not only the physical properties but also the chemical properties (e.g., oxidative property) of PUFA-GPLs mediate their functions. One example is ferroptosis, which is a form of cell death triggered by peroxidized PUFA-GPLs and is investigated as a potential anti-cancer strategy (94).

### Roles of membrane PUFAs

Many functions of PUFA-GPLs were unveiled through LPLAT research (Table 1) (73–76, 95–103). AGPAT3-deficient mice, which have reduced DHA-GPLs, display spermatogenesis defects and retinal dysfunctions (74, 75). Indirect evidence suggests that DHA-GPLs, by reducing bending rigidity, promote the formation of strongly bent membranes during spermatogenesis (for the removal of cytoplasm by Sertoli cells by tubulobulbar complex) and during maturation of rhodopsin-containing retinal disc membranes, although the role of DHA-derived lipid mediators (collectively termed docosanoids) in these processes is also possible.

LPCAT3-deficient mice have specific reductions in GPLs containing AA (AA-GPLs) and are neonatally lethal due to enterocyte dysfunction and malnutrition, which are triggered by overaccumulation of triglycerides in the cytosol derived from mother’s milk (73, 100). Liver-specific LPCAT3 deficiency leads to hepatic steatosis under a high-fat diet (100). These phenotypes do not match with those of known eicosanoid receptor- or eicosanoid synthetic enzyme-deficient mice, and basal eicosanoid levels are unchanged in tissues from LPCAT3-deficient mice (73). This suggests that AA-GPLs, and not eicosanoids, are important for triglyceride clearance from enterocytes and hepatocytes. Indeed, triglycerides surrounded by AA-GPLs are better transported by microsomal triglyceride transfer protein, which is critical for secretion of triglycerides and lipoprotein production (73). Therefore, the phenotypes of LPCAT3-deficient mice revealed previously unrecognized functions of PUFA-GPLs at the interface between the cytosol and triglycerides surrounded by endoplasmic reticulum leaflets. Further studies revealed that PUFA-PCs reduce the surface tension at the interface between water and triglycerides and affect the budding of lipid droplets in vitro (104). It will be interesting to investigate the relationship between this observation and the phenotypes of LPCAT3-deficiency. In addition to these triglyceride-related phenotypes, LPCAT3 deficiency in intestinal stem cells causes the overproduction of cholesterol, which further leads to cellular overproliferation (103). This overproliferation enhanced tumor formation in an Apc^min^ genetic background (a mouse model of colon cancer). While the implication of sterol regulatory element-binding protein 2 was shown in this process, detailed molecular mechanisms connecting LPCAT3, AA-GPLs, and cholesterol regulation remain to be solved. A role of LPCAT3 in adipocyte differentiation was also reported (96). Using 3T3-L1 cells as a model, knockdown of LPCAT3 by shRNA decreased PUFA-GPLs (note that in contrast to other reports, DHA-GPLs also changed) and inhibited adipogenesis. The Wnt/β-catenin pathway was activated upon LPCAT3 knockdown, the inhibition of which restored adipogenesis. This makes a potential link between AA-GPLs and adipogenesis through Wnt/β-catenin. However, regulatory roles of eicosanoids in adipogenesis have also been documented (105), thus it remains to be established whether AA-GPLs regulate the process.

MBOAT7 deficiency leads to defects in neuronal migration and neurite outgrowth, leading to abnormal brain morphology in mice (76). Rare mutations in human MBOAT7 also cause neurodevelopmental defects (106). MBOAT7 deficiency decreases AA levels not only in PI but also in phosphoinositides (76). Quantitative changes in total levels of phosphoinositides are also reported (95). It is therefore possible that aberrant signaling by phosphoinositides is involved in the outcomes of MBOAT7 deficiency, but effector proteins that can discriminate phosphoinositide acyl-chains remain to be discovered. In line with this possibility, genetics studies in *Caenorhabditis elegans* suggest that PI acyl-chains affect PI 3-phosphate signaling (98). Genetic variants of MBOAT7 in humans are reported to affect nonalcoholic fatty liver disease, but inconsistencies are also suggested (107). In mice, liver-specific MBOAT7 knockdown or knockout promotes fatty liver (108, 109). Triglyceride synthesis is promoted under MBOAT7 insufficiency, in part due to the higher turnover of PI (both synthesis and degradation are promoted) and the higher production of diacylglycerol thereof (see “PI cycle” in Fig. 1) (110). It will be interesting to investigate whether phosphoinositides are also involved in the fatty liver phenotype.

The investigation of LPLATs also revealed how PUFA-GPLs affect the functions of membrane proteins. By combining PUFA feeding and genetic manipulation of LPLATs in *Caenorhabditis elegans*, it was found that PUFA-GPLs regulate proteins involved in touch sensation (110). In another study, researchers generated transgenic worms expressing human TRPV4 (transient receptor potential vanilloid 4) to study how PUFA-GPLs affect its function (111). Through a combination of PUFA feeding and manipulation of LPLATs in these worms, they demonstrated a regulatory role of PUFA-GPLs (more specifically, GPLs containing PUFA epoxides) in vasodilation. These studies demonstrate the usefulness of LPLATs as a tool to discriminate the functions of PUFAs as lipid mediators or as membrane components.

### Future directions

Although it was difficult to discriminate whether PUFAs execute their functions through PUFA-derivatives or PUFA-GPLs, LPLAT research will probably help with this discrimination and make the functions of PUFA-GPLs clearer. It remains challenging to discover their precise molecular mechanisms, but many useful tools are being developed, such as proteome-wide identification of PUFA-interacting proteins (112), and genome-wide knockout screens (113). It is also important to visualize localization and movement of PUFA derivatives in the cells. Combination of these tools with the manipulation of PUFA-GPLs using LPLATs will be a promising approach to achieve a deep understanding of PUFA-GPL functions. Finally, the existence of PUFA level sensors is suggested in multiple studies (110, 114), which will be important to identify. With all of these issues and others cleared, we will finally have a clear understanding about how PUFAs affect health and disease.
